# 

**Published:** 1832-12

**Authors:** 


### MONTHLY LIST OF MEDICAL BOOKS.

[*Medical Works cannot be entered on this List unless a copy be sent for the purpose, the titles of Books having frequently been sent to us as published which have not appeared for weeks, or even months, after.*]

The Elements of Anatomy. By Jones Quain, B.M., Professor of Anatomy and Physiology in the University of London. Second Edition.—8vo. pp. 812. John Taylor, London.

A correct, well-arranged, and perspicuously written system of Anatomy. Its value as a book of reference is greatly increased by a copious alphabetical index.

The Cyclopædia of Practical Medicine. Edited by John Forbes, M.D., A. Tweedie, M.D., and John Conolly, M.D. Part XI. (published in Monthly Parts,) November 1832,) Containing *Hydrophobia* (continued), by Dr. Bardsley; *Hydrothorax*, Dr. Dandell; *Hypertrophy*, Dr. Townsend; *Hypertrophy of the Heart*, Dr. Hope; *Hypochondriasis*, Dr. Prichard; *Hysteria*, Dr. Conolly; *Ichthyosis*, Dr. A. T. Thomson; *Identity*, Dr. Montgomery; *Impetigo*, Dr. A. T. Thomson; *Impotence*, Dr. Beatty; *Incontinence of Urine*, Dr. Cumin; *Incubus*, Dr. Williams; *Indigestion*, Dr. Todd.—Royal 8vo. pp. 127. Sherwood, Gilbert, and Piper; and Baldwin and Cradock, London.

Medico-Chirurgical Transactions, published by the Medical and Chirurgical Society of London. Vol. XVII.—8vo. pp. 513; plates. Longman and Co., London.

A Dictionary of Practical Medicine: comprising General Pathology, the Nature and Treatment of Diseases, Morbid Structures, and the Disorders especially incidental to Climates, to the Sex, and to the different Epochs of Life: with numerous Prescriptions for the Medicines recommended, a Classification of Diseases according to Pathological Principles, a copious Bibliography, with References, and an Appendix of approved Formulæ. The whole forming a Library of Pathology and Practical Medicine, and a Digest of Medical Literature. By James Copland, M.D., Consulting Physician to Queen Charlotte's Lying-in Hospital, &c. Part I.—8vo. pp. 352. Longman and Co., London.

A brief Outline of the History and Progress of Cholera at Hull; with some Remarks on the Pathology and Treatment of the Disease. By James Alderson, M.D., late Fellow of Pembroke College, Cambridge, &c.—8vo. pp. 38; plates. Longman and Co., London.

A Lecture on the Nature, Causes, and Prevention of Cholera, delivered at the Ivanhoe Bath's Assembly Rooms, Ashby-de-la-Zouch, in aid of the Funds of the Cholera Provident Society; and published at the particular request of the Board of Health of that place. By J. M. S. Kennedy, M.D.—8vo. pp. 36. Sherwood and Co., London.

Part XII. and XIII. The Principles and Practice of Obstetric Medicine, in a Series of Systematic Dissertations on Midwifery and on the Diseases of Women and Children. Illustrated by numerous Plates. By D. D. Davis, M.D., M.R.S.L., Professor of Midwifery in the University of London, &c.—John Taylor, London.

An Essay on the Structure and Functions of the Skin; with Observations on the Agency of Atmospheric Vicissitudes, through the medium of the Skin, in the Production of Affections of the Lungs, Liver, Stomach, Bowels, &c. By Wm. Wood, M.D., Member of the Royal College of Surgeons in London, &c.—8vo. pp. 172. Baldwin and Cradock, London; and Maclachlan, Edinburgh.

A Treatise on Inflammations; explaining their Pathology, Causes, Consequences, and Treatment, with their Effects on the various Textures of the Body. Being an Extension of a "Dissertation on Inflammation of the Membranes," to which the Jacksonian Prize for 1828 was awarded by the Royal College of Surgeons in London. By George Rogerson, Surgeon, of Liverpool. Vol. I.—8vo. pp. 459. Longman and Co., London.

### APOTHECARIES' HALL.

NAMES OF GENTLEMEN to whom the Court of Examiners have granted Certificates :

NOV. 1.

William Rowland, of Wrexham
George Smith, Barnsley

NOV. 8.

Henry Alexander
John Joseph Ayre, Lynn, Norfolk
William Coward, North Shields
William Hanrot Cory, Yarmouth
James Shepherd Darby
James Gatis, Lynn, Norfolk
John Hodgson, Chepstow
John Whisler Jex, Lynn, Norfolk
John Mitchell, Kingston, Herefordshire
Samuel Nicholson, London
John Ryan, Shoreditch
William Wilkins, Bath

NOV. 15.

James Brooking, Plymouth
Thomas Beales, Hopton, Suffolk
Samuel Gauntlett, Olney
Garland Foley Bailey Harrison, Devonport
John Hedges Marshall, Wallingford
John Spencer Pidcock, Alstonfield
Edward Rendle, Plymouth
Robert Smith, Chirley
Daniel Saunders, Redbourn
Stephen Yeldham, Halstead, Essex

NOV. 22.

Henry Chorley, Leeds
Thomas Peacock.

### INDEX

TO THE

### THIRTEENTH VOLUME OF THE NEW SERIES

OF

### THE LONDON MEDICAL AND PHYSICAL JOURNAL.

---

PAGE

**Abdomen**, injection thrown into, after tapping, caused death . . 517
Acarus, description and engraving of a new . . . . . 523
Agues treated by holly and ilicine . 89
Air in respiration, on the alterations of 501
Amenorrhœa cured by leeches to the mammæ . . . . 256
Anatomy Bill passed . . 172
—, Act for regulating schools of, &c. . . . . . 345
Anesthæsia, case of . *(foot note)* 137
Aneurism by anastomosis, cases of . 250
—, cases of popliteal; hemorrhage on nineteenth day; pulsation in, on twelfth day . . 512
Aphonia, cases of, depending upon head affection . . . . 266
Apothecaries' Hall: names of gentlemen admitted . 88, 175, 264, 352, 440, 528
— Company, their regulations for students . . . 434
Arachnitis, symptoms, &c. of . 143
Argentum nitratum recommended in burns, &c. . . . 429
Asphyxia, death from . . 520
Assafœtida, in large doses, serviceable in hysterical vomiting and neuralgia 336
—, remarkable effect of smell of, upon wolves . . 171
Australian plants, on some medicinal products of . . . 275

Barry, Alex., Esq. F.R.S., death of 438
Beulah Spa, at Norwood . . 154
Birth, six children at one . 522
Blood, on the sources and nature of the powers on which the circulation of, depends . . . 214
Bones, on diseases of the . 139
Botany, Professor Burnett's lecture on, 8, 111, 285, 376, 466
—, review of works on . 402
Breech presentations, management of 301
British Association for the Advancement of Science . . 172
Burnett, Professor, lecture on Botany, 8, 111, 285, 376, 466
Burns, on the treatment of . 144, 219

PAGE

Burns, use of Arg. Nit. in . 429

Capillaries, on the influence upon the motion of the blood in the, from the action of the heart . . 216
Carbonic acid, suffocation by . 278
Carotid artery divided, and voluntary locomotion to a considerable distance afterwards . . 430
Chiritmanos of Peru . . 373
Cholera, notices of various works on 329
—, treatment of, at Darlington 334
—, official paper on . 84
—, increased weight of the atmosphere on the occurrence of the epidemic . . . . 176
—, proof from insurance companies that the disease was confined to the lower orders . . 261
—, origin, symptoms, &c. . 413
—, use of purgative neutral salts in, 273
—, influence of animal and vegetable matters upon . . 433
—, account of the injection of large quantities of saline solutions into the veins . . . 77
—, case of the contagious propagation of . . . 155
—, proofs of its being contagious 428
—, notice of Dr. Webster's essay on 151
—, cherry-laurel water in . 340
—, nature and treatment of . 416
—, remarks on . . 5
—, surgeon fined for not reporting cases of . . . 261
Choleraphobia, cases of . 341
Choleric diathesis, test of . . 171
Civilization, influence of, upon man 398
Cold as a cause of disease . 21, 198
—, effects of, on the respiratory function . . 21
— on animal heat . 22
— on the vascular system 24
— on absorption . 29
— on the nervous system ib.
—, diseases of . . 198
—, circumstances most favorable to, as a morbific agent . 203
—, forms of, most dangerous . 207

Cold, prevention of diseases from . 209

Conception influenced by the period of the year . . . 391

Convulsions, puerperal . . 306

Convulsive disease in infants . 320

Croton oil, effects of, used externally and internally . . . 253

Cystocele, fatal case of . . 52

Delirium from revulsion of erysipelas 252
Dew, oily appearance of . . 344
Dogwood, Jamaica, on the medical properties of . . . 177
Dropsy, case of, in which a radical cure was attempted by throwing an irritating injection into the abdomen, with a fatal result . . . 517

Effluvia . . . 395

Egyptian pupils, account of their arrival, with M. Clot, in Paris . 525

Egypt, on some ancient plants of 368

Emanations . . . 393

Emphysema, cases of a new species of, after hemorrhage . . 505

occurring in a patient after the application of leeches . 159

Epidemic, remarkable, consisting of mortification of the thumb . 252

Erysipelas, hairpowder applied in . 183

, revulsion of, causing delirium, which was cured by the return of inflammation . . 252

Famine, account of the, in Guzerat 258
Fingers, on retraction of . 510
Fœticide, on the laws of England as they relate to . . . 184
Fœtus, on spontaneous amputation of the limbs of, in utero . 162

Ganglia of the nerves, use of the . 443
Gangrene, M. Dupuytren on various forms of . . . 421
Genital organs, cases of amputation of 508
Glottis, spasm of the, in infants . 320
Grotta del Cane, account of . 278

Hairpowder applied in erysipelas . 183
Head, injuries of the, involving important questions in medical jurisprudence 164
Hearing, on the physiology of the organ of . . . . 240
Heart, spontaneous rupture of . 517
Heat of young animals . . 493
Hemorrhage, emphysema following 505
Hernia, case of strangulated femoral 49
, case of strangulated umbilical 45
of bladder, fatal case of . 52
Hip, on dislocations of the, by Baron Dupuytren . . . 66
Home, Sir Everard, Bart., death of 351
Holly in the cure of intermittent fevers 89
Horse, Mr. Percivall's work on the anatomy of the . . . 416
Hydrocephalus, chronic, cured by puncture . . . 161, 256
Hydrophobia from the bite of a fox 65
Hygiène, views of . . 388
Hysterical vomiting and neuralgia cured by large doses of assafœtida . 336

Ilicine in the cure of intermittent fevers 89
Infanticide, remarks on . 353
, value of the hydrostatic test in . . . . 356
Inflammation, review of Mr. James' work on . . . 314
Insects, influence of, on vegetation 8
Intermittent fevers, on holly and ilicine in the cure of . . . 89
Irritability and respiration, on the inverse ratio which exists between, in the animal kingdom . . 406

Larva of a small species of Cytiscus 59
Lime, on the use of chloride of, to destroy the smell of paint . . 258
Lithospermum officinale, analysis of the stony pericarp of . . 260

Malaria, on the effects of . . 441
Medical practitioners cannot be obliged to certify as to the state of a hospital patient without a fee . . 524
Medicines, action of, upon the nervous energy . . 479
, absorption of, through the lungs . . 482
, their effects on the living solids . . 483
, circumstances modifying the action of . . 485
, custom modifies the action of 486
, climate modifies the action of ib.
, mental affections, influence of, on the action of . 489
, their action influenced by the period of disease. . ib.
Medulla spinalis, recovery after wound of the . . . . 137
Microscope, improvements of the 223
Microscopes with diamond and jewel lenses . . . . 57
Midwifery, half-yearly report of cases of 110
Mortality influenced by the season 391

Navy Medical Board, regulations of the, for assistant surgeons . . 437
Nephritis arising without obvious cause 419

Peru, on the Chiritmanos of . 373
Phlegmasia dolens in the male . 247
Phthisis, case of, cured; tubercle expectorated . . . . . 516
Physical agents, their influence on life 492
Piscidia Erythrina, on the medical properties of . . . 177
Placenta, on the structure of the: opinions of the Hunters questioned by Dr. R. Lee . . . 71
Plants, ancient, of Egypt . 368
Poisoning by sulphuric acid . 1
effected by mineral acids; no reliance to be placed upon analysis of the contents of the stomach . 4
Polypus, reproduction of, when divided 62
Portal, Baron, biographical sketch of 261
Pregnancy, case assuming appearances of 309
, awful death in last month of 313
, in bar of execution, ought to be admitted whether the woman has *quickened* or not . . 197
, on detection of, by the state of the urine . . 127
Prostate gland, cases of abscess of . 337
Pruritis of the generative organs in females cured by nitrate of silver 126
Puerperal fever, remarks on . 129
Pupa, transformation of, to the perfect fly . . . . 61

Quickening, remarks upon the loose application of the term . 190

Regulations for students by the Apothecaries' Company . . 434
of the College of Surgeons for candidates for a diploma . ib.
Respiration and irritability, on the inverse ratio which exists between, in the animal kingdom . , 406
Revulsion, on the laws of . 33
Rickets, on the treatment of . 139
Ringworm propagated by contagion 161

Sap in plants, on the motion of the 224, 227
Scalds, use of Arg. Nitratum in . 429
Shoulder presentation, management of 305
Skull, compound fracture of . 425
Spasm of the glottis in infants . 320
Spina bifida . . . 131
, cases of . . 133
Spina bifida, on puncturing the tumor 134
Spine, concussion of . . 135
, injury of the, causing anomalous symptoms . . 136
fractures of the, sometimes recovered from . . 137
, injuries of . . 131
Suffocation by carbonic acid . 278
Sulphuric acid, poisoning by . 1
Sulphur, on the formation of, at the Solfatara . . . 343
Sympathies, Sabatier's divisions of 38

Tic douloureux cured by stramonium 252
Trades, effects of various, on health and longevity . . 232
Tubercle expectorated in a case of phthisis . . . . 516
Tumor of an uncommon nature in the biceps muscle . . . 55
Turning, on the obstetric operation of 167

Urethra, different opinions concerning the structure of the . . 44
Uterine hemorrhage avoided in women who are predisposed to it . 476
Uteri, prolapsus . . . 107
Uterus, inflammation of unimpregnated 126
, retroversion of the, during pregnancy . . 341
, spontaneous inversion of the 128

Vagina, imperforation of the, successfully treated . . . 246
Valley of death, visit to the . 522
Veins, large quantities of saline solutions thrown into the, in cases of cholera 77
Vomiting during pregnancy, prussic acid in . . . 127

---

COLLECTANEA . . 71, 160, 252, 340, 428, 516
INTELLIGENCE . . 77, 172, 260, 345, 433, 524
METEOROLOGICAL REGISTER 88, 176, 264, 352, 440, 528

### CRITICAL ANALYSES.

Les Lois de la Revulsion, etudiées sous le Rapport Physiologique et Therapeutique. Par J. C. Sabatier (d'Orleans), Docteur en Médecine de la Faculté de Paris . . 33

Observations in Surgery and Pathology, illustrated by Cases and by the Treatment of some of the most important Surgical Affections. By W.J.Clements, Surgeon . . . 43

The Microscopic Cabinet of select Animated Objects, with a Description of the Jewel and Doublet Microscopes, Test Objects, &c. To which are subjoined, Memoirs on the Verification of Microscopic Phenomena, and an exact Method of appreciating the Quality of Microscopes and Engiscopes, by C. R. Goring, M.D. By A. Pritchard 56

An Introduction to the Practice of Midwifery, by the late Thomas Denman, M.D. Seventh Edition, with additional modern Information on the various Subjects treated of in the Work, and a Dissertation on the Transfusion of Blood in the more dangerous varieties of Uterine Hemorrhage, &c. By Chas. Waller, M.D. &c. . . 125

A Treatise on the Injuries, the Diseases, and the Distortions of the Spine. By R. A. Stafford, Esq. . . 131

Two Lectures on the Primary and Secondary Treatment of Burns. By Henry Earle, F.R.S. . 144, 219

On the Sources and Nature of the Powers on which the Circulation of the Blood depends. By A. P.W. Philip, M.D. &c. 214

Improvements in the Microscope, by Mr. C.Varley and Mr. W. Valentine; with a supplementary Letter from R. H. Solly, Esq. on certain Parts of Vegetable Structure . . 223

The Effects of Arts, Trades, and Professions, and of Civic States and Habits of Living, on Health and Longevity. Second Edition, greatly enlarged. By C. T. Thackrah, Esq. . . 232

Practical Observations on Midwifery, with a Selection of Cases. Part II. By John Ramsbotham, M.D. . 300

Observations on the General Principles and on the particular Nature and Treatment of various Species of Inflammation. By J. H. James, Esq. 313

On Spasm of the Glottis in Infants. By Dr. W. B. Joy . . . 320

The Sources of Health and Disease in Communities, or Elementary Views of Hygiène. By Henry Belinaye, Esq. 388

Organographie Végétale, par M. Aug. Pyr. De Candolle . . 402

Conversations on Vegetable Physiology ib.

Physiologie Végétale, par M. Aug. Pyr. De Candolle . . . ib.

An Introduction to Botany, by John Lindley, F.R.S. L.S. G.S. &c. . ib.

Theory on the Inverse Ratio which subsists between the Respiration and Irritability in the Animal Kingdom. By Marshall Hall, M.D. F.R.S. L. & E. 406

Elements of Materia Medica and Therapeutics, including the recent Discoveries and Analyses of Medicines. By A. T. Thomson, M.D. F.L.S. & G.S. &c. 478

On the Influence of Physical Agents on Life, by W. F. Edwards, M.D. F.R.S. Translated from the French, by Dr. Hodgkin and Dr. Fisher. To which are added, in the Appendix, some Observations on Electricity; on Absorption, and the uses of the Spleen; on the Microscopic Characters of the Animal Tissues and Fluids; and some Notes to the Work of Dr. Edwards 492

### BIBLIOGRAPHICAL NOTICES.

A Practical Treatise on Uterine Hemorrhage, in connexion with Pregnancy and Parturition. By John T. Ingleby, Esq. M.R.C.S. &c. . . 63

An Essay on the Epidemic Cholera. By John Webster, M.D. . . 151

Practical Observations on the Medicinal Virtues of the Beulah Saline Spa, Norwood, &c. By A. Maxwell, Esq. 154

The Anatomy and Physiology of the Organ of Hearing, by David Tod, Esq. 240

Clinical Reports of the Surgical Practice of the Glasgow Royal Infirmary, by John Macfarlane, M.D. . . 245

A Simple and Effectual Plan for the Prevention of the Malignant Cholera. By John Burne, M.D. . 329

Observations on Spasmodic Cholera, by Henry M'Cormac, M.D. . . 330

A Letter on the Treatment of Cholera, by John French, Esq. . . 332

A Treatise on Cholera, by J. Keir, M.D. 333

Experimental Inquiries in Chemical Pathology. Part I. By H. Prater, Esq. 334

Stephenson and Churchill's Medical Botany. Edited by Gilbert T. Burnett, F.L.S. &c. . . . 412

Essay on the Origin, &c. of Cholera Morbus, by T. Foster, M.B. . 413

The Anatomy of the Horse, by W. Percivall, M.R.C.S. . 416

Nature and Treatment of the Epidemic Cholera, by R. Venables, A.M. &c. 416

Dissertation upon a new Species of Emphysema, developed after abundant Hemorrhage. By M. E. Rebolle de Gex . . . . 505

### NAMES *of* AUTHORS *of whose Works, Observations, &c. a more or less detailed Account is given in this Volume.*

Amos, Professor, case shewing the possibility of Voluntary Locomotion to a considerable distance, after the Division of the Carotid, &c. . . 430
Andral, Professor, on the Effects of Croton Oil, used externally and internally . . . . 253
Barry, Alexander, Esq. F.R.S., account of his death . . . 438
Belinaye, Henry, Esq., review of his work on Hygiène . . 388
Blake, Andrew, M.D. M.R.C.S., on the Effects of Malaria, &c. . . 441
Blondin, M., case of furious Delirium from Revulsion of Erysipelas . 252
Bollaert, W. Esq., account of the Chiritmanos of Peru, and of the Medicines sold by them . . . 373
Bonastre, M., on the ancient Plants of Egypt . . . . 368
Brault, Dr. L., case of contagious Cholera 428
Bright, Dr., case of Phlegmasia Dolens in the Male . . . 247
Burne, John, M.D., notice of his Plan for the Prevention of Cholera . 329
Burnett, Professor, Lecture on Botany, 8, 111, 285, 376, 466
Carnac, Capt. J. R., account of the Famine in Guzerat in 1812 and 1813 258
Clements, W. J. Esq., review of his Observations in Surgery . . 43
Clendinning, John, M.D., on Cold as a Cause of Disease . . 21, 198
Clot, M., his arrival in Paris, with his Egyptian Pupils . . 525
Cox, J. C. F.L.S. &c., on the use of the Arg. Nitratum in Burns . . 429
Collineau, M., case of Propagation of Ringworm by Contagion . 161
Cusack, Mr., case of Fracture of the Skull 425
Dance, Dr., case of Nephritis, arising without obvious cause . . 419
De Candolle, M. A. P., review of his Organographie Végétale, and his Physiologie Végétale . . 402
Desmée and Gendron, MM., case of complete Imperforation of Vagina, successfully treated . . 246
case of Emphysema, occurring in a Consumptive Patient after the application of Leeches on the Chest . . . . 159
Dudon, Dr., Cherry-laurel water in Cholera . . . . 340
Dupuy, M., case in which an irritating Injection into the Abdomen caused Death . . . . . 517
Dupuytren, M., on certain forms of Gangrene . . . . 421
on Dislocations of the Hip 66
on Removal of the Genitals 50[illegible]
on Retraction of the Fingers . 510
Dwyer, Mr., case of Hysterical Vomiting and Neuralgia, cured by very large doses of Assafœtida . . . 336
Earle, Henry, F.R.S., Lectures on Burns 144, 219
Edwards, W. F., M.D. F.R.S., on the Influence of Physical Agents in Life 492
Forster, Mr., account of a remarkable Epidemic . . . 252
, T., M.B. F.L.S. &c., notice of his Essay on Cholera . . 413
Fott, Dr., Tic Douloureux cured by Stramonium . . . 252
French, J. G. Esq., notice of his Letter on Cholera . . . 329
Goring, C. R. M.D., notice of his Microscopic Cabinet of select Animated Objects, &c. . . . 56
Graefe, Professor, case of Chronic Hydrocephalus cured by Puncture . 160
Hall, Marshall, M.D. F.R.S. L. & E. &c., on the Inverse Ratio between the Respiration and Irritability in the Animal Kingdom . . . 406
Hamilton, Wm. M.B., on the Medical Properties of the Piscidia Erythrina, or Jamaica Dogwood . . 177
Heming, G. O. Esq., on Prolapsus Uteri 107
James, J. H. Esq., review of his Treatise on Inflammation . . 313
Ingleby, J. T. Esq., notice of his Treatise on Uterine Hemorrhage . 63
Joy, Dr. W. B., review of his article in the Cyclopædia of Medicine, "on Spasm of the Glottis in Infants" 320
Keir, Dr. J., notice of his Treatise on Cholera . . . 329
Key, Dr., on the Prevention of Uterine Hemorrhage . . . 476
Laennec, M., case of Phthisis cured, in which a Tubercle was expectorated 516
Lee, Robert, M.D. F.R.S., on the Structure of the Human Placenta , 71
Le Hunte, Captain C., analysis of the Lithospermum officinale . 260
Lewins, R. M.D., on Saline Injections in Cholera . . . . 79
Lindley, John, F.R.S. L.S. G.S. &c., review of his Introduction to Botany 402
Loudon, A. Esq., Visit to the Valley of Death, in the Island of Java . 522
Loudon, C. M.D., on the Cure of Amenorrhœa by Leeches to the Mammæ 256
M'Cormac, H. M.D., notice of his Observations on Cholera, &c. . 329
Macfarlane, Dr., cases of Abscess of the Prostate . 337
cases of Aneurism 512
Treatment of Cholera at Darlington . 334
notice of his Clinical Reports of the Surgical Practice of the Glasgow Royal Infirmary . 245

Macfarlane, Dr. J., cases of Aneurism by Anastomosis . . 250
Maxfield, A. Esq., notice of his Observations on the Beulah Spa, Norwood 154
Montgomery, W. F., M.D. M.R.I.A., on the Spontaneous Amputation of the Limbs of the Fœtus in Utero, &c. 162
Mudie, Mr., Observations on some Medicinal Products of Australian Plants 275
Peacock, Dr., Treatment of Cholera at Darlington . . . 334
Percivall, Wm., M.R.C.S. &c., notice of his Anatomy of the Horse . 416
Philip, A. P. W., M.D. F.R.S. L. & E., review of his Paper on the Sources and Nature of the Powers on which the Circulation of the Blood depends 214
Portal, Baron, Biographical Sketch of, with an account of his death . 261
Prater, Horatio, notice of his Experimental Inquiries in Chemical Pathology . . . . 329
Pritchard, A. Esq., notice of his edition of Goring's Microscopic Cabinet of select Animated Objects . . 56
Radford, T. Esq., Observations on the Obstetric Operation of Turning . 167
Ramsbotham, Dr. John, review of his Observations in Midwifery, Part II. 300
Rebolle de Gex, M. E., on a new Species of Emphysema following Hemorrhage 505
Ridgway, Dr. T., on Cholera . 5
———— on the use of the Purgative Neutral Salts in Cholera . 273
Rolland, M., Retroversion of the Uterus during Pregnancy . . 341
Rousseau, Dr. L. F. E., on the use of Holly and Ilicine in the cure of Intermittent Fevers . . . 89
Russel, R. C. Esq., case of Chronic Hydrocephalus successfully treated by Puncture . . . 256
Sabatier, Dr. J. C., review of Les Lois de la Revulsion . . . 33
Solly, R. H. Esq., on certain Parts of Vegetable Structure . . 227
Somerville, Dr. J. C., his Regulations in regard to Anatomy . . 349
Stafford, R. A. Esq., review of his Treatise on Diseases, &c. of the Spine 131
Stephenson and Churchill's Medical Botany, edited by G. T. Burnett, F.L.S. &c., notice of . . . 412
St. Vincent, Bory de, Description of a new Acarus . . . 523
Taylor, A. S. Esq., an account of the Grotta del Cane, with Remarks on Suffocation by Carbonic Acid . 278
———— case illustrative of the Contagious Propagation of Cholera 155
———— case of Poisoning by Sulphuric Acid 1
———— Remarks upon Infanticide . . 353
———— on the Formation of Sulphur at the Solfatara, near Naples 343
———— Remarks on the Law of England as it relates to the crime of Fœticide , . . 184
Tellier, Dr., cases of Choleraphobia, &c. 340
Thackrah, C. T. Esq., on the Effects of Arts, Trades, &c. on Health and Longevity . . . 232
Thomson, A. T. M.D. &c., review of his work on Materia Medica and Therapeutics . . . . 478
Tod, David, Esq., notice of his work on the Anatomy and Physiology of the Organ of Hearing, &c. . . 240
Townsend, Dr. R., case of Death from Asphyxia . 525
———— case of spontaneous Rupture of the Heart 517
Varley and Valentine, Messrs., on Improvements in the Microscope . 223
Venables, Robert, A.M., notice of his Essay on Cholera . . 416
Waller, Dr. C., half-yearly Report of Cases in Midwifery 110
———— review of his edition of Denman's Midwifery 125
Watson, A. Esq. F.R.C.S. E., Medico-legal Examination of two Cases of Death from Injuries of the Head, involving important questions in Medical Jurisprudence . . . 164
Webster, Dr. John, cases of Aphonia depending upon an Affection of the Head . . . . 265
———— notice of his Essay on Cholera 151

ADLARD, PRINTERS, BARTHOLOMEW CLOSE.

